# Supplementary material for: Inducing Mitotic Catastrophe as a Therapeutic Approach to Improve Outcomes in Ewing Sarcoma
Source: Cancers (Basel). 2023 Oct 10;15(20):4911. doi: 10.3390/cancers15204911 (PMC10605681; doi:10.3390/cancers15204911)
Supplement: Supplementary file 1 [file cancers-15-04911-s001.zip › Supplementary Figures proofread ST.pptx]

## Slide 1
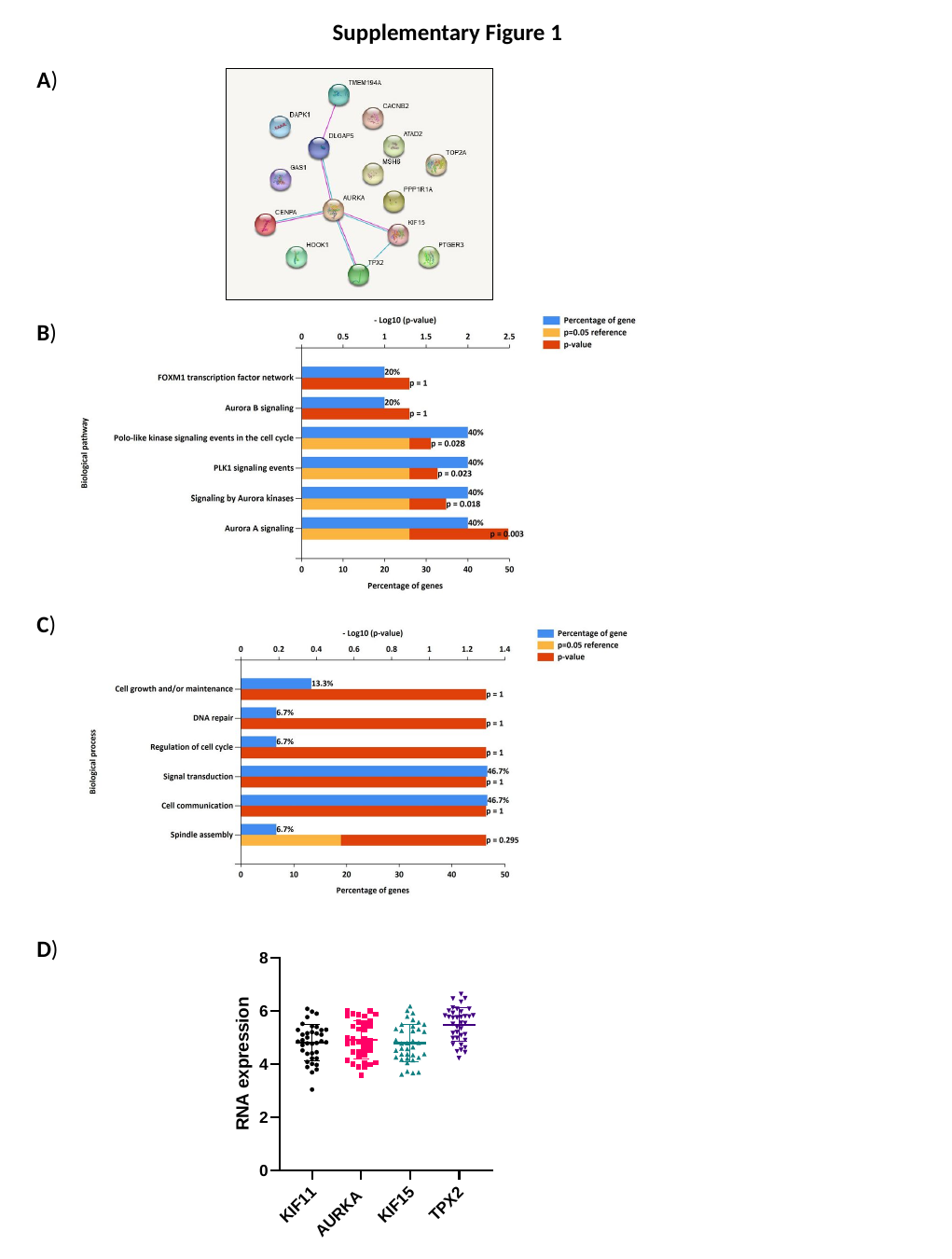

Supplementary Figure 1
A)
B)
C)
D)

## Slide 2
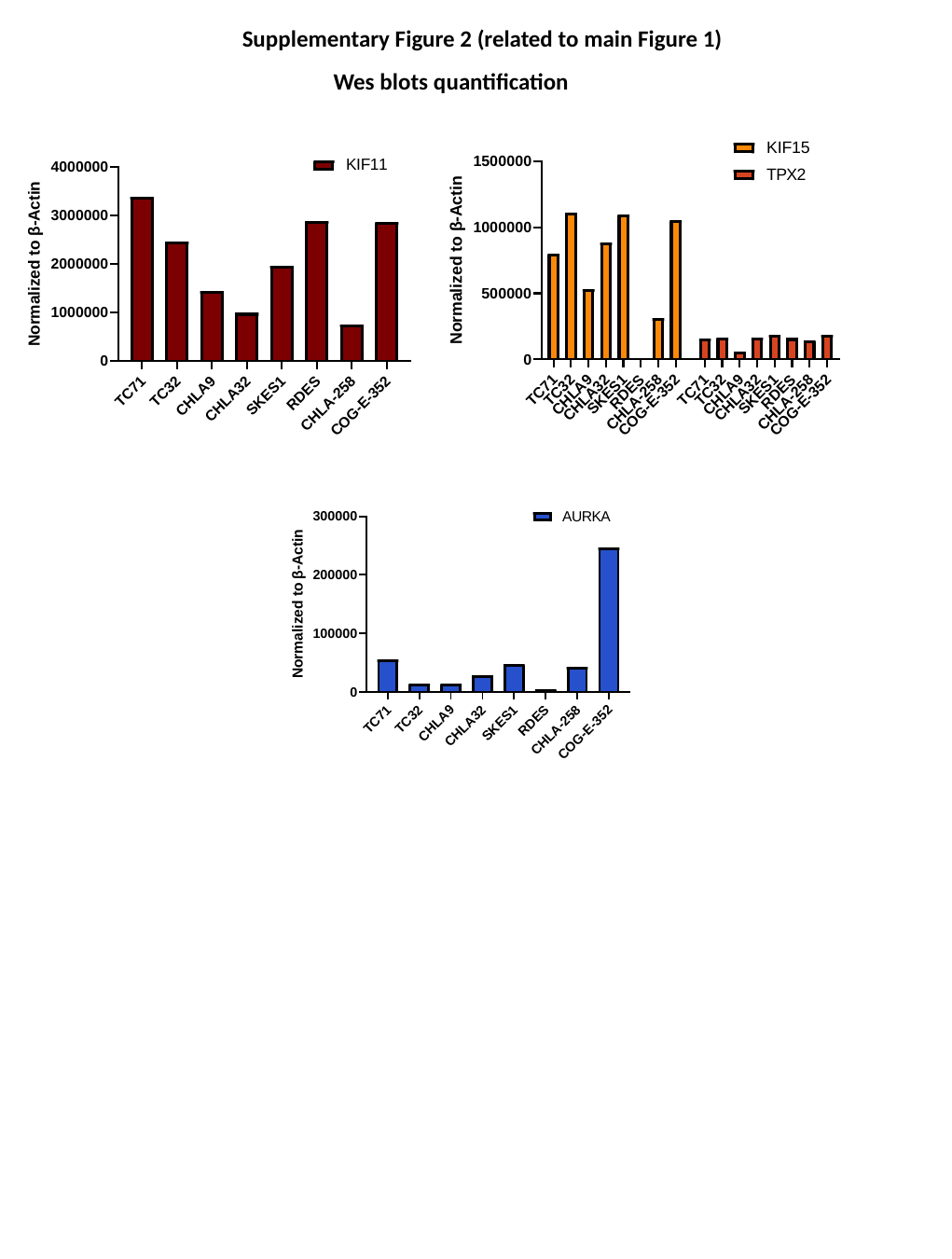

Supplementary Figure 2 (related to main Figure 1)
Wes blots quantification

## Slide 3
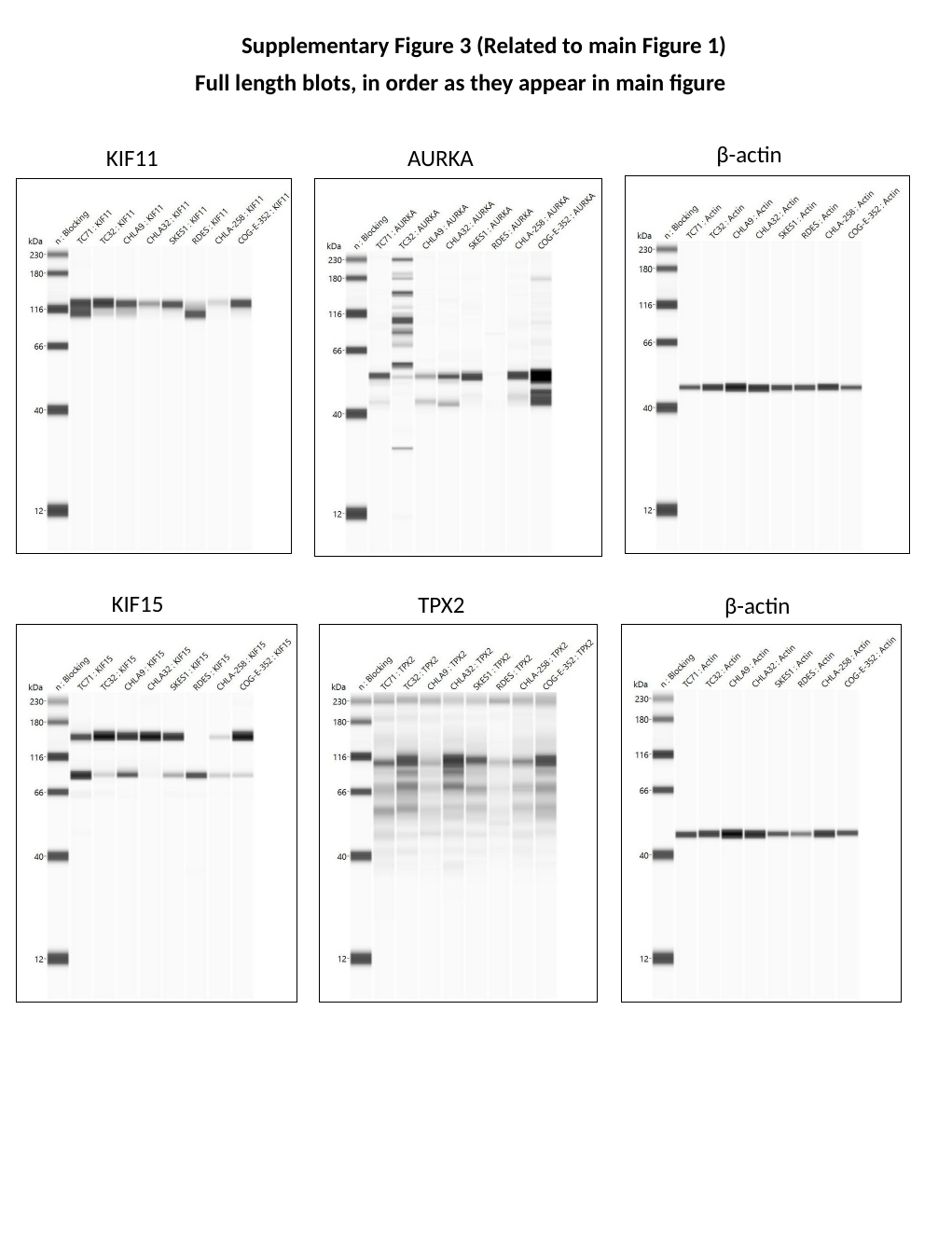

Supplementary Figure 3 (Related to main Figure 1)
Full length blots, in order as they appear in main figure
β-actin
KIF11
AURKA
KIF15
TPX2
β-actin

## Slide 4
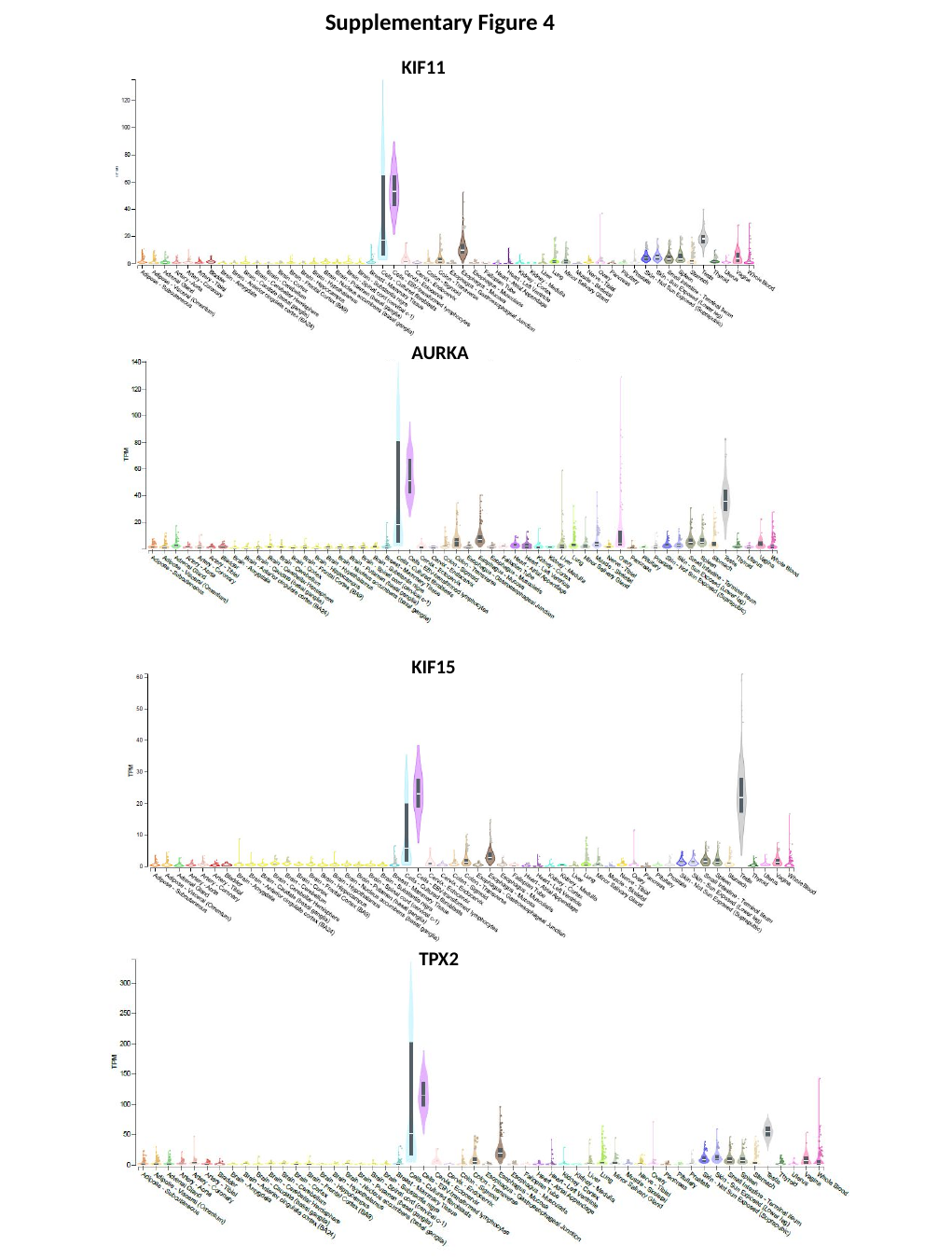

Supplementary Figure 4
KIF11
AURKA
KIF15
TPX2

## Slide 5
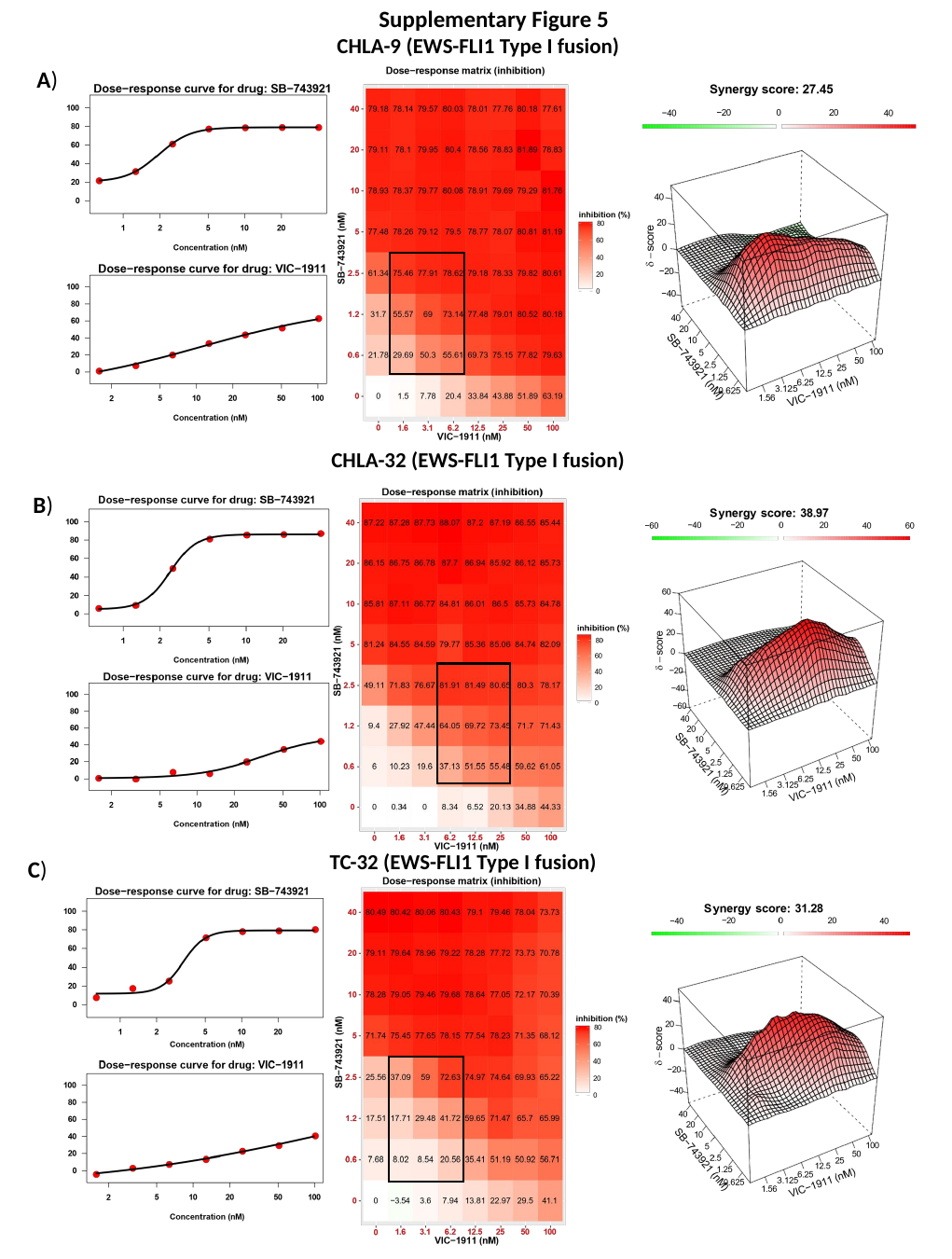

Supplementary Figure 5
CHLA-9 (EWS-FLI1 Type I fusion)
A)
CHLA-32 (EWS-FLI1 Type I fusion)
B)
TC-32 (EWS-FLI1 Type I fusion)
C)

## Slide 6
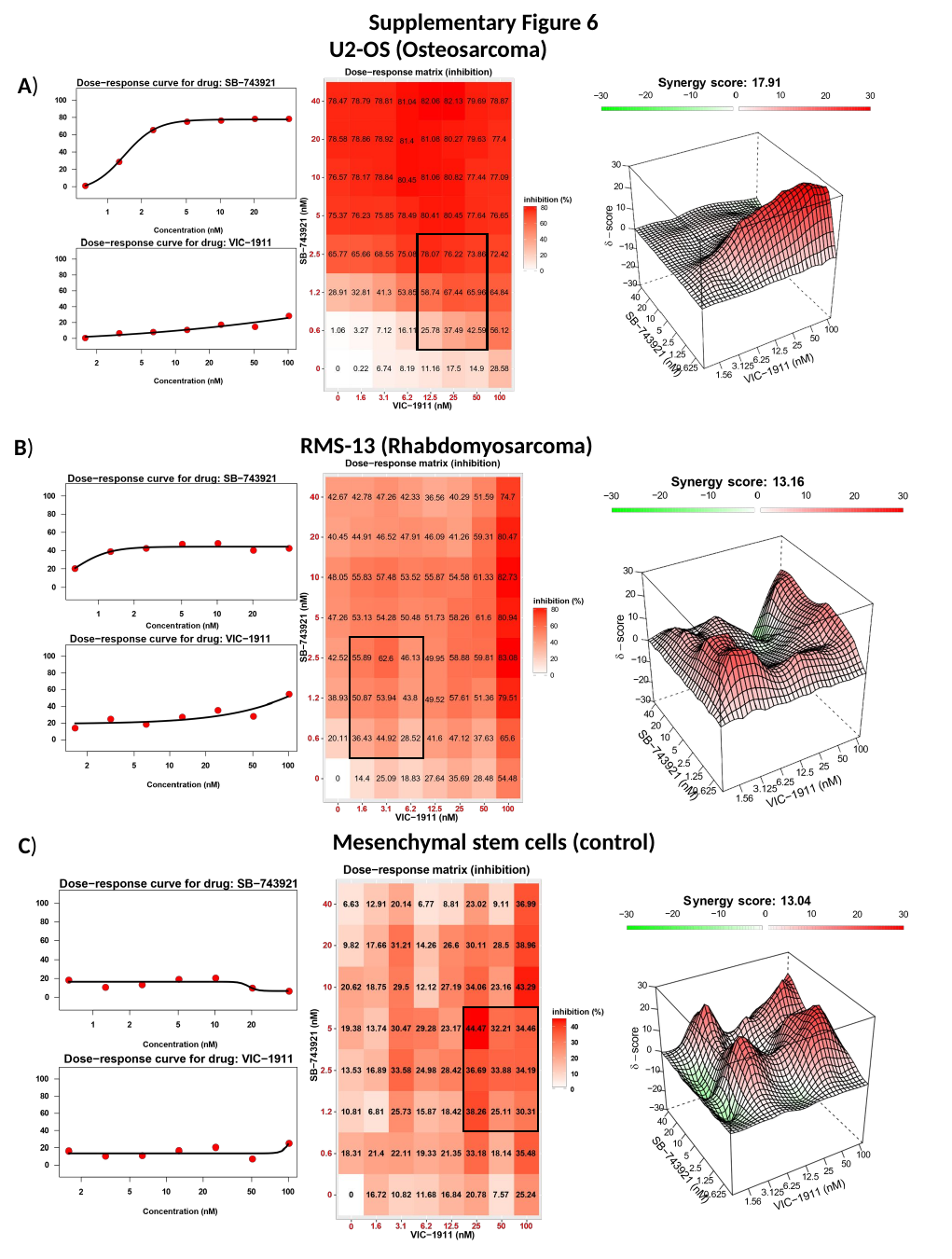

Supplementary Figure 6
U2-OS (Osteosarcoma)
A)
RMS-13 (Rhabdomyosarcoma)
B)
Mesenchymal stem cells (control)
C)

## Slide 7
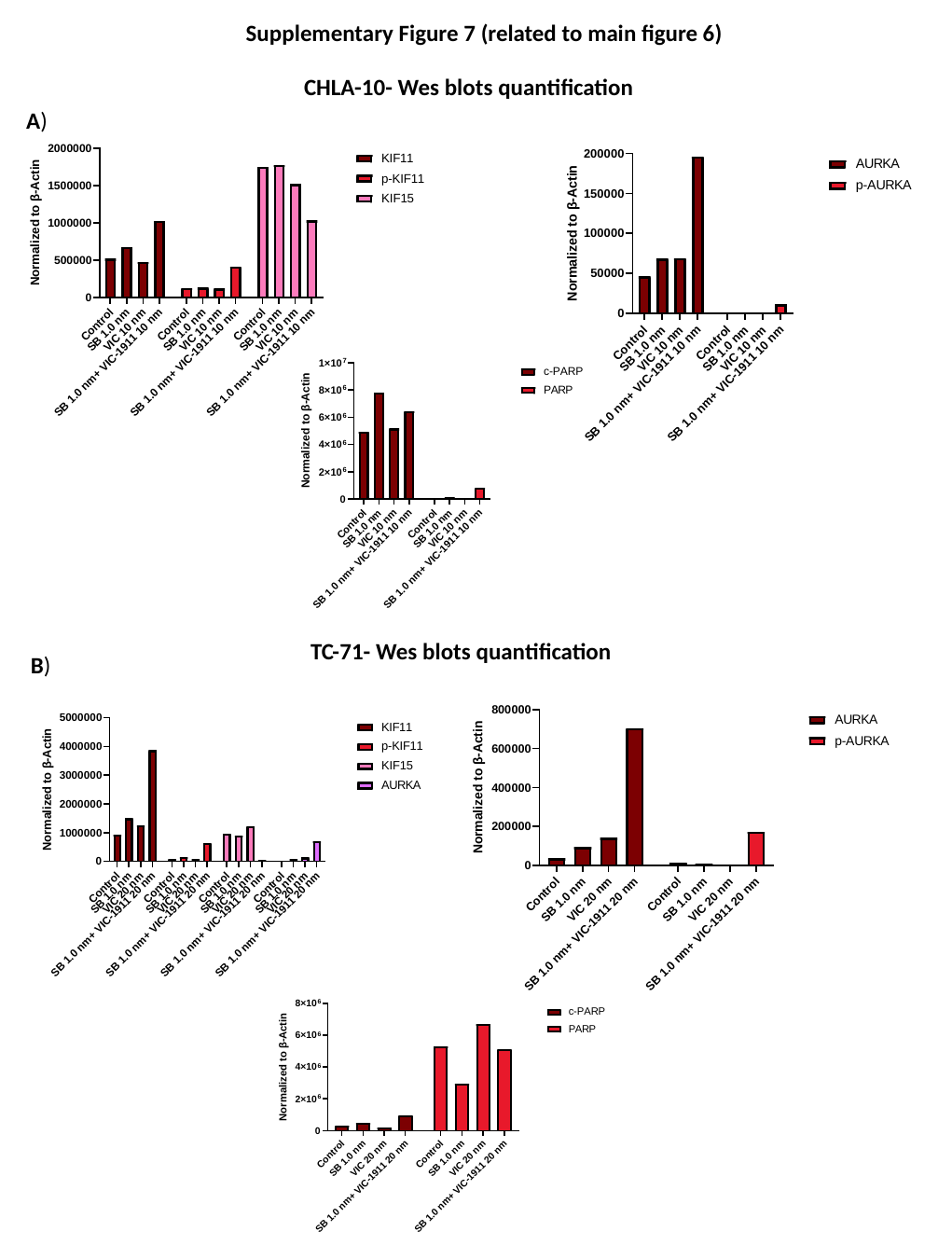

Supplementary Figure 7 (related to main figure 6)
CHLA-10- Wes blots quantification
A)
TC-71- Wes blots quantification
B)

## Slide 8
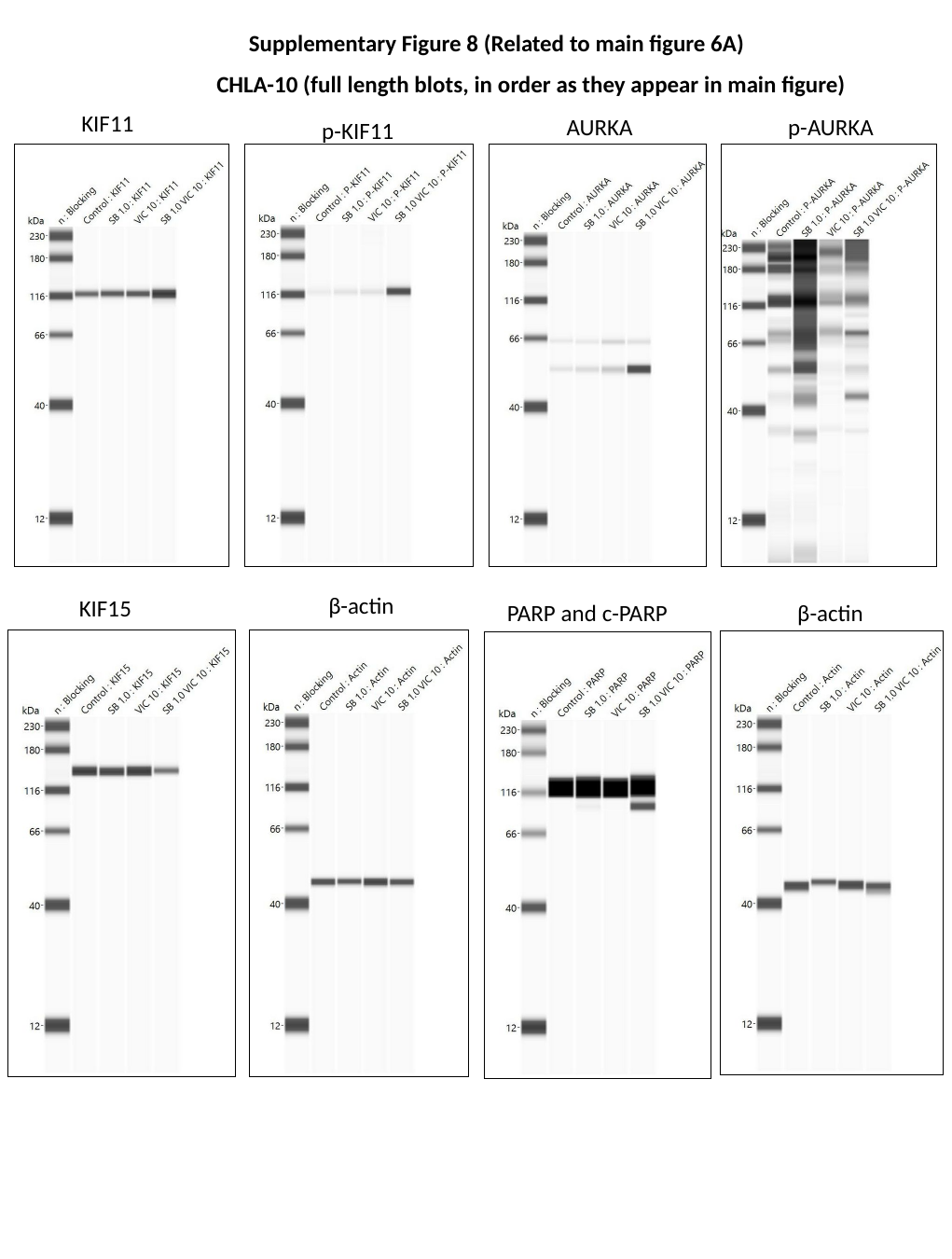

Supplementary Figure 8 (Related to main figure 6A)
CHLA-10 (full length blots, in order as they appear in main figure)
KIF11
AURKA
p-AURKA
p-KIF11
β-actin
KIF15
PARP and c-PARP
β-actin

## Slide 9
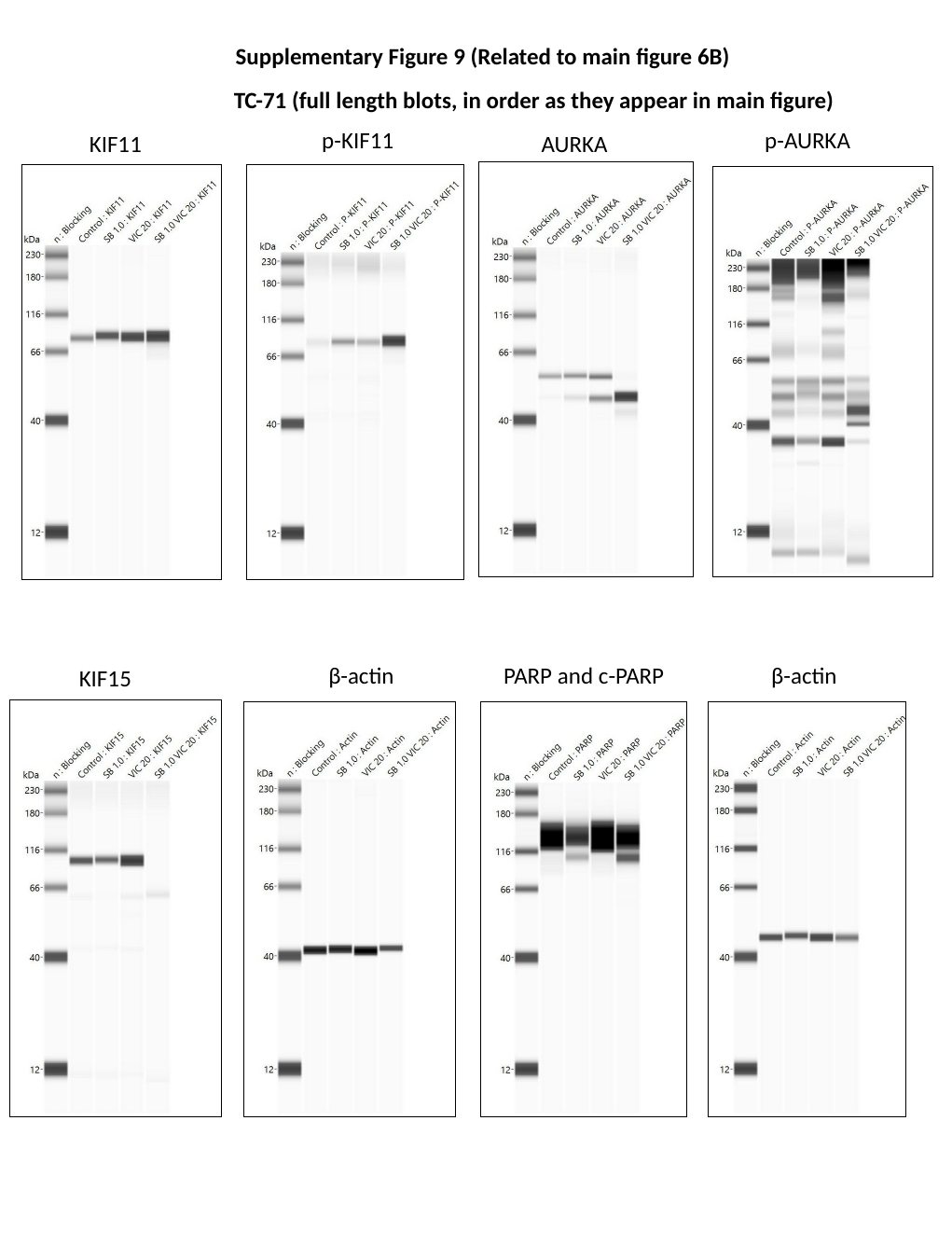

Supplementary Figure 9 (Related to main figure 6B)
TC-71 (full length blots, in order as they appear in main figure)
p-AURKA
p-KIF11
AURKA
KIF11
β-actin
β-actin
PARP and c-PARP
KIF15

## Slide 10
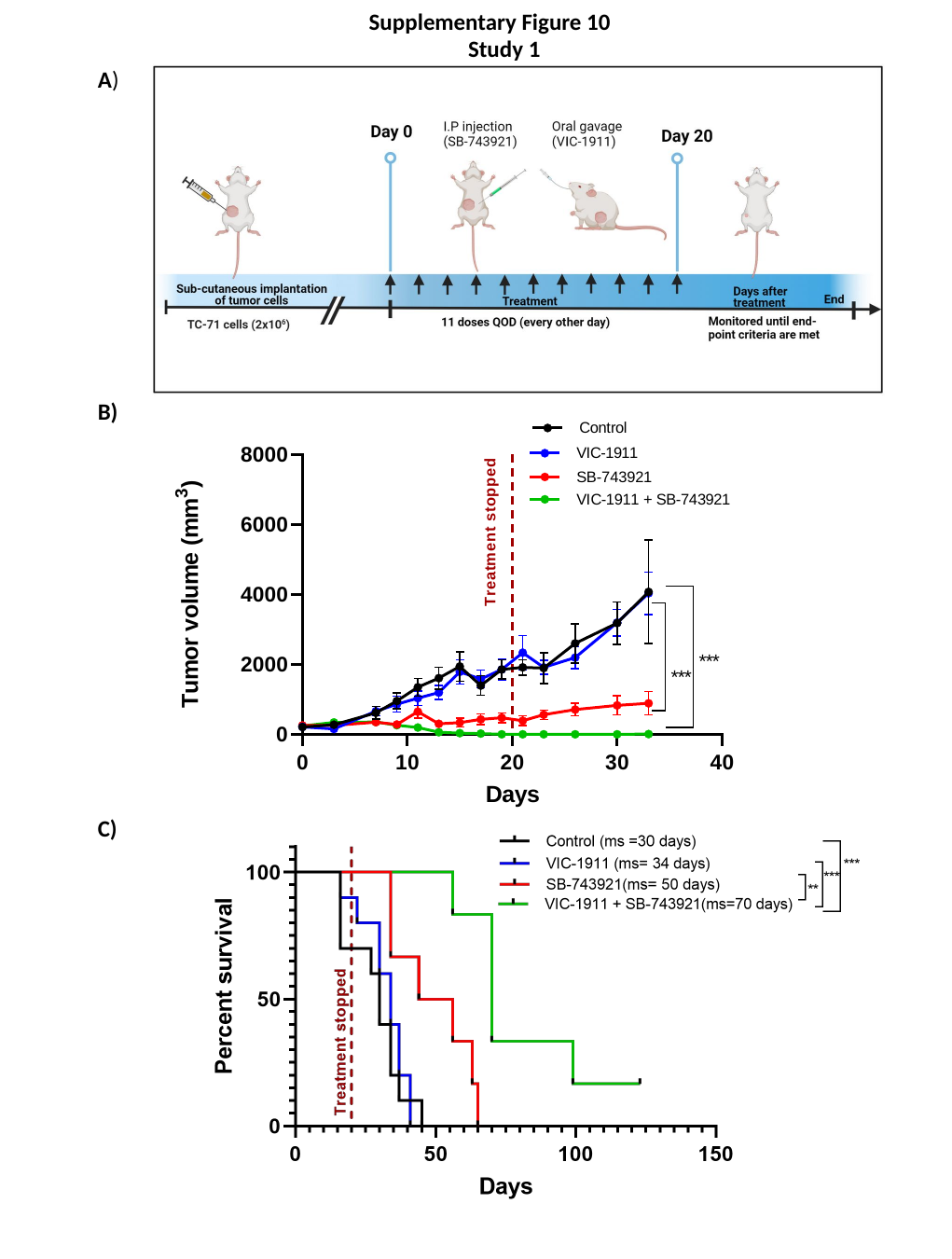

Supplementary Figure 10
Study 1
A)
B)
C)

## Slide 11
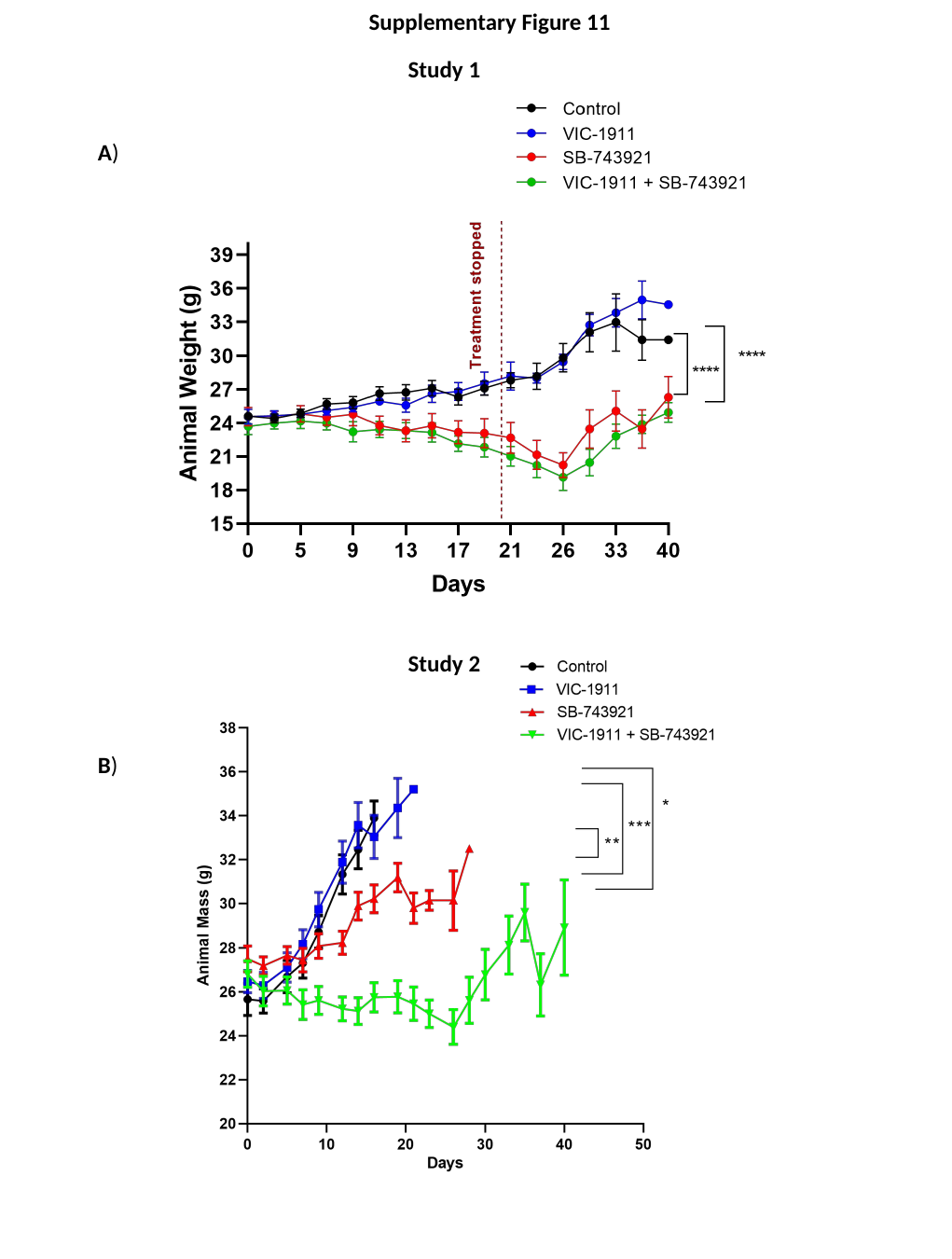

Supplementary Figure 11
Study 1
A)
Study 2
B)
