# Supplementary material for: Inducing Mitotic Catastrophe as a Therapeutic Approach to Improve Outcomes in Ewing Sarcoma
Source: Cancers (Basel). 2023 Oct 10;15(20):4911. doi: 10.3390/cancers15204911 (PMC10605681; doi:10.3390/cancers15204911)
Supplement: Supplementary file 1 [file cancers-15-04911-s001.zip › Supplementary Legends proofread ST.pdf]

SUPPLEMENTARY FIGURE LEGENDS

Supplementary Figure S1. A) STRING protein-protein interaction analysis for the top-fifteen genes is represented. Gene enrichment analysis of the top-fifteen genes by FunRich 3.1.3 (Functional Enrichment Analysis Tool) software indicating the key **B**) biological pathways, and **C**) biological processes mediated by these genes. **D**) RNA expression of KIF11, AURKA, KIF15 and TPX2 from EWS patients were accessed from BioGPS.

Supplementary Figure S2 (related to Figure 1B & C). Quantification data of Wes blots.

Supplementary Figure S3 (related to Figure 1B & C). Full length Wes blots.

Supplementary Figure S4. Gene expression data for KIF11, AURKA, KIF15 and TPX2 from The Genotype-Tissue Expression (GTEx) portal (https://gtexportal.org/home/) accessed on 3 October 2023**.**

Supplementary Figure S5. Dose-response curves, dose-response matrix, and heat map indicating synergy scores in different EWS cell lines. Combination treatment resulted in the following synergy scores in **A**) CHLA-9 (27.45), **B**) CHLA-32 (38.97), and **C**) TC-32 (31.28).

Supplementary Figure S6. Dose-response curves, dose-response matrix, and heat map indicating synergy scores in different cancer cell lines and control. Combination treatment resulted in the following synergy scores in **A**) U2-OS (17.91), **B**) CRL-2061 (13.16), and **C**) BMD-MSCs - control (13.04).

Supplementary Figure S7 (related To Figure 6A & B). Quantification of Wes blot data for **Figure 6** is shown.

Supplementary Figure S8 (related To Figure 6A). Full length Wes blots.

Supplementary Figure S9 (related To Figure 6B). Full length Wes blots.

Supplementary Figure S10. A) Timeline of in vivo study is represented. SB-743921 (2.5 mg/kg) and VIC-1911 (37.5 mg/kg) were dosed intraperitoneally and orally respectively**.** Mice were treated with these drugs every other day for 20 days (11 treatments) and monitored until end-point criteria were met. We observed significant **B**) tumor regression (control vs combination and VIC-1911 vs combination, ***$p \leq 0.001$ as assessed by one-way ANOVA) and **C**) overall survival. ***$p \leq 0.001$ and **$p \leq 0.01$ is determined by logrank (Mantel-cox) test and indicated on the graph.

Supplementary Figure S11. The body weights of mice are represented for the tumor efficacy **A**) efficacy Study 1 and **B**) efficacy Study 2. ****$p \leq 0.0001$, ***$p \leq 0.001$, **$p \leq 0.01$ and *$p \leq 0.05$ as measured by one-way ANOVA.
